# “Sometimes You Get Good Ones, and Sometimes You Get Not-so-Good Ones”: Vendors’ and Consumers’ Strategies to Identify and Mitigate Food Safety Risks in Urban Nigeria

**DOI:** 10.3390/foods11020201

**Published:** 2022-01-12

**Authors:** Stella Nordhagen, James Lee, Nwando Onuigbo-Chatta, Augustine Okoruwa, Eva Monterrosa, Elisabetta Lambertini, Gretel H. Pelto

**Affiliations:** 1 Global Alliance for Improved Nutrition (GAIN), 1202 Geneva, Switzerland; emonterrosa@gainhealth.org; 2 Independent Researcher, Toronto, ON M6G 2K4, Canada; leejam17@yahoo.com; 3 Global Alliance for Improved Nutrition (GAIN), Abuja 900103, Nigeria; nonuigbo-chatta@gainhealth.org (N.O.-C.); aokoruwa@gainhealth.org (A.O.); 4 Global Alliance for Improved Nutrition (GAIN), Washington, DC 20036, USA; elambertini@gainhealth.org; 5 College of Human Ecology, Cornell University, Ithaca, NY 14853, USA; gp32@cornell.edu

**Keywords:** foodborne illness, food choice, ethnographic research, traditional markets, sociocultural beliefs and practices, food safety attitude, knowledge and behavior

## Abstract

This paper uses detailed data from in-depth interviews with consumers (*n* = 47) and vendors (*n* = 37) in three traditional markets in Birnin Kebbi, Nigeria. We used observations from those markets to examine how consumers and vendors identify and avoid or manage food safety risks and whom they hold responsible and trust when it comes to ensuring food safety. At the level of the vendor, consumers mentioned seeking “clean” or “neat” vendors or stalls. Cleanliness was primarily related to the appearance of the vendor, stall, and surroundings; reliance on trusted, known vendors was also noted. Food products themselves were largely evaluated based on visual cues: insects, holes, and colors—with some reliance on smell, also. Similarly, vendors assessed safety of food from suppliers based on a visual assessment or reliance on trusted relationships. On the second research question, both consumers and vendors largely placed responsibility for ensuring food safety on government; when asked specifically, consumers also named specific steps that vendors could take to ensure food safety. Consumers and vendors also generally felt that they could limit many food safety risks through identifying the “good” products in the market or from suppliers. The paper discusses the implications of these results for behavior change interventions.

## 1. Introduction

Foodborne illness is estimated to be responsible for a disease burden comparable to malaria, contributing to about half a million deaths annually [1], particularly in low- and middle-income countries (LMICs) [2,3]. In addition to harming health, unsafe food comes with a large economic price, estimated at about $20 billion USD per year, due to sickness, loss of life, treatment costs, and trade impacts [4]. Food safety is defined as the assurance that food will not cause harm to the consumer when prepared or eaten according to its intended use [5]. Contaminants that can make food unsafe include foodborne pathogens (viruses, bacteria, protozoa), parasites, chemical contaminants, naturally occurring and biogenic toxins, or adulterants). Risk of contamination can increase in transitioning lower-middle-income countries, like Nigeria: rapid economic, demographic, and dietary change lead to increased exposure (e.g., through longer supply chains and more food consumed outside the home), while government (and private-sector) capacity to manage food safety remains limited [4]. Unfavorable food safety conditions can be particularly acute in traditional markets, which often have poor infrastructure [6], imperfect hygiene and storage conditions [7,8], and limited oversight [9,10], but are of great importance as a food source to consumers in LMICs [11].

Indeed, food safety risks are widespread in Nigeria, including through contamination, adulteration, misbranding, illegal trading, inadequate export and import controls, and no or inappropriate training of food handlers [12,13]. A recent review found high levels of bacterial contamination at the point of sale in vegetables (up to 60% of samples), fruits (up to 52%), beef (up to 22%), pork (up to 30%), dairy (up to 10%), and fish (100% in one study) [12,14]. It is thus unsurprising that the country has a high burden of foodborne illness, with an estimated 33,000 deaths annually and annual treatment costs of approximately USD 1.7 billion [12].

In high-income countries, food safety risks are usually managed by actors throughout the supply chain, with considerable oversight from governmental food safety system functions such as permitting, inspections, and detection of and response to illness outbreaks [15,16]. Most LMICs, however, struggle to implement and enforce similar approaches due to a lack of resources to support public food safety systems, as well as a lack of resources for private businesses to consistently implement risk control measures [4,17]. In Nigeria, existing policies cover all stages of the food supply chain and both domestic and export markets but are imperfectly implemented, with little coordination among agencies and inadequate inspection and surveillance [13]. In a recent assessment, over 95% of relevant local government authorities were found unable to undertake adequate collection, analysis, and dissemination of food safety data; few had adequate systems to respond to outbreaks; and almost no labs could adequately investigate food contaminants [18].

In the absence of functioning control systems, mitigation of food safety risk falls to supply chain actors such as vendors and to consumers. Indeed, studies elsewhere have documented that consumers and vendors have strategies they use to mitigate food safety risks, such as buying from trusted sources [19,20]. Most research in Nigeria and other LMICs, however, used a top-down approach, focusing on whether vendors (and, to a lesser extent, consumers) follow standard expert-agreed-upon practices to minimize food safety risks (such as cleaning utensils or washing surfaces) [21]. It has rarely aimed to obtain a “bottom-up” understanding of how consumers and vendors may (or may not) devise their own strategies to guide decisions related to food safety. Indeed, it is rare in food safety research worldwide to examine local perspectives in-depth, including through qualitative methods [22]. This is despite evidence that experts and non-experts often have widely different perceptions related to food safety [23,24,25]. Among consumers, research in LMICs has also generally focused on in-home food handling practices (e.g., handwashing) as opposed to decisions made in the market [26]. 

Designing appropriate behavior change approaches to reduce food safety risk, however, requires understanding what strategies people already use—to build upon them where helpful and correct them where not [4,22]. This paper thus examines what consumers and vendors actually do about food safety, using their own words and conceptions, when needing to make choices within a system that cannot guarantee that food available for purchase is safe. It does so through a study of consumers and vendors in three traditional markets in Birnin Kebbi, a mid-sized city in northwestern Nigeria. 

The research questions are:

1. How do consumers and vendors identify and avoid or manage food safety risks in a market setting?
2. Whom do vendors and consumers hold responsible and trust when it comes to ensuring food safety in market settings?

The results are discussed in the context of prior research on similar topics in other settings, to draw implications for the design of behavior change interventions focusing on food safety in markets in Nigeria and other LMICs. 

## 2. Materials and Methods

A companion paper [27] examined the food safety beliefs of consumers and vendors in the same setting, considering how they conceive of food safety and whether it was a driver of their choices. The methods are provided in detail there and are only summarized briefly here. 

This study used an adapted Focused Ethnographic Study (FES) approach [28,29], which entails two iterative phases. The initial phase examined key themes and was followed by rapid analysis and discussion with the research team to identify emerging topics of interest, which were examined in depth through a second phase. It engaged vendors and consumers in three markets in Birnin Kebbi. It focused on seven ‘focus foods’: rice, maize, cowpea, soybean, fish, green leafy vegetables (GLV; including spinach, moringa, pumpkin leaves, cabbage, and other greens; normally cooked before eating), and beef, chosen due to local priorities, nutritional value, widespread consumption, and covering a diverse range of food types.

Fieldwork, conducted by trained local interviewers in February–May 2021, consisted of in-person interviews with consumers and vendors as well as a structured observation in each market. Respondents were recruited through market visits. To be eligible, consumers needed to shop in at least one target market, at least once a month, and have primary or shared responsibility for purchasing food for their household. Quotas were set for the following consumer characteristics: men, women, those under age 30, those over age 30. Vendors needed to sell at least one focus food in a target market at least once a month. In Phase 1, three vendors each for beef, fish, and GLV were chosen (as these foods were thought to have the highest associated food safety risk) and one vendor each for soybean, maize, rice, and cowpea was interviewed. In Phase 2, based on priorities identified in Phase 1, only GLV and fish vendors were interviewed (12 of each). The sample size for consumers was 16 in Phase 1 and 31 in Phase 2; for vendors, it was 13 and 24, respectively. 

The research followed standard ethical procedures, as well as local COVID-19 protocols, and all participants provided signed informed consent. Interviews were audio recorded in Hausa, then transcribed and translated into English. Quantitative data were analyzed in Excel and Stata SE15, while transcripts were analyzed through hand-coding or using the qualitative data software ATLAS.ti. All quotations in the text are presented verbatim, aside from small corrections to typos and punctuation, and annotated with a de-identified code for the respondent.

## 3. Results

Demographic information for the consumers and vendors is given in Table 1 and Table 2, respectively. Consumers include a mix of men and women, with the typical respondent being in their thirties, Hausa, Muslim, and married with children. Of note, the sample is fairly well educated and affluent, with half of respondents having completed tertiary education and only 12% of households estimated as being below the 3.10 PPP per person, per day international poverty line according to the Poverty Probability Index [30]. All of the ‘focus foods’ were purchased regularly by all or nearly all consumers, with the exception of soybean, which was purchased less often (in some cases, never) and usually for specific uses, such as feeding young children. Traditional markets were the primary source used for fresh foods (such as the focus foods) for all consumers. 

Though the methods originally called for interviewing both female and male vendors, no eligible female vendors were identified for the majority of the foods studied. Interviewed vendors are thus all men. The typical vendor is around age 40 and a Hausa Muslim who may have completed primary school but likely is not educated at a higher level. About a third of vendors sold foods in addition to the ‘focus foods’, in particular, grain/legume vendors tended to sell multiple types of grains/legumes and GLV vendors often sold other vegetables. 

### 3.1. The Traditional Market as a Setting

The observations revealed numerous challenges in keeping food safe within a market setting (Table 3). All three markets sell multiple food types, but in only one market were these clearly divided into different sections by food type (in the others, fresh vegetables and animal-source foods could be sold on adjacent tables, increasing risk of cross-contamination). Of the three studied markets, only one had piped water and toilets. None had formal garbage collection facilities; instead, there was an unstructured open space in which garbage was dumped and occasionally burned. One had concrete flooring, while the others were dirt-floored; most vendors used wooden (as opposed to concrete) tables, and some had no table or stall whatsoever. All markets also had informal mobile sellers clustered outside the market, who largely sold from the ground. The smallest market was noted by the observer to be generally unclean, due to the sandy ground, dirt blowing around, litter, hovering flies, and “a strong whiff of decaying fish in the air.” None of the markets had prices routinely displayed for products; instead, the price was communicated by the vendor to the client and could be bargained over. Most observed transactions between vendors and shoppers were said to be “quick and business-like.”

### 3.2. How Do Consumers Avoid Unsafe Foods?

As noted in [27], interviewed consumers are somewhat aware of food safety issues and have some experience with foodborne disease, but this is not an overriding concern; considering food safety, their main worries are related to chemical contamination (pesticides, herbicides, fertilizers, and preservatives) and linked to cowpea, GLV, and beef. 

#### 3.2.1. Choosing a “Clean” Vendor

The main actions consumers took to ensure they acquired safe food began at the step of choosing a vendor within a market. While some did mention cleanliness as playing a role in the initial choice of deciding which market to visit, market choice was driven by other factors: prices, breadth of products, convenient location, and habit. Once choosing a vendor, however, more than half of consumers noted criteria related to food safety—namely, cleanliness—as being one factor considered. This was particularly true in the second phase of the study, when questions on vendor choice appeared after those discussing food safety as a health issue, suggesting some effect of framing.

*I have one to two vendors that I buy things from, **some of them look dirty and you won’t like to interact with such a person, so you go to another person**. Some are neat but their goods are pricey… things are expensive, but one will still want a seller that you trust will not cheat you.–C1207, a 31-year-old female consumer*

*[emphasis added]*

*There are people who are more hygienic than the others. There are some products I buy from my vendor, and **there are products I buy from other people because of their hygienic level.**–C1209, a 40-year-old male consumer*

*[emphasis added]*

Men were particularly likely to mention cleanliness as a factor. The main things that respondents mentioned regarding vendor and shop cleanliness were: cleanliness of the table (e.g., washed, no dirt/blood); neatness of the table or wares (e.g., arranged in neat stacks); cleanliness of the vendor; cleanliness of the surroundings; cleanliness of the food (e.g., visible dirt on food or packaging); presence of insects; and proper food handling. A few also mentioned goods being covered or stored in containers or not being stored on the ground, one noted lack of a smell, and another noted good flooring.

*[At the market], the only thing I look out for is when the place is clean. When the place is neat and very attractive, I’ll get what I want to get. If the environment and shop is unkempt, sometimes I doubt things from them, but it is clean and neat, I get things from there…. The person too should be clean… The outfits, and the neatness of the person, the personal hygiene of the person: you don’t go and meet with the person… [if he] looks as if he is from a mechanic workshop.–C2201, a 25-year-old male consumer*

*[To feel confident in the foods I buy, I look at] how he [the vendor] handles those stands, does he normally sweep… does he wash those bowls that he’s using …there are some people even the bowl will be up to 100 years, they will not wash it! They don’t mind how it’s being taken care of, what they are concerned about is money, how they will get money. So I’m being concerned about those things.–C2210, a 35-year-old female consumer*

*Some of them [the vendors], this spinach when they bring it, they just keep it on the ground and that ground is not a clean environment… when they keep it on the ground people step on it and also the ground it dirty, after picking it they will not wash it they will just pick it and put it on top of the table, so that spinach has already be contaminated with disease on the ground.–C2211, a 40-year-old female consumer*

#### 3.2.2. Recognizing Unsafe Foods

Integrated with the choice of a vendor was the choice of a specific food product. As food safety issues and food traits vary widely across food types, this was examined through the specific focus foods, with a focus on beef, GLV, and fish (foods associated with higher risk according to experts, [12]). Most respondents cited specific signs they could use to identify safe products. However, some felt that there was little or nothing they could do to identify safe foods, expressing a degree of fatalism about the need to live with the risk, that the nature of a market was to have variable quality, or that the only thing to be done was to stop eating the food:

*Sometimes you get good ones, and sometimes you get bad ones* *. Every day is not Sallah [Eid al-Fitr] or Christmas day. That’s a market for you.–C2204, a 40-year-old female consumer*

For cowpea, the food for which consumers had the most food safety concern, weevils were detected through physical damage and contamination (holes and weevils being visibly present), while chemicals were detected via a bad smell.

*Sometimes when I go to the market to buy beans [cowpea] and I noticed the beans has many holes on it or weevils, I don’t buy it. I prefer to buy something good…. I think when one buys beans that are weevil infested, it is very possible that after eating, it will cause one to be stooling. [Also] when I notice the chemicals in beans is too much, I don’t buy it. Because when the chemical is too much, it has bad effect on the beans.–C1205, a 53-year-old male consumer*

For rice, consumers were worried about the presence of stones in local rice varieties (which were harvested and processed using minimal mechanization). These were hard to detect visibly at the point of purchase, so customers instead reported using trusted vendors and/or buying foreign industrially processed rice, which was thought to be free of stones.

*I have one [vendor], I always prefer buying rice from him because he is also very honest. Anytime I want to buy ‘stone free’ rice, he has always given me the assurance that what he will give me has no stones…. So, once I get home, I will be expecting to hear complaints about the rice after cooking it, like the normal complaints when I buy rice that has stones, but to my surprise, no one will have anything bad to say about the rice.–C1211, a 38-year-old male consumer*

For maize and soybean, concerns over food safety were very limited, and respondents noted that they took no steps to avoid unsafe versions. In contrast, beef was associated with food safety issues by many consumers—second only to cowpea as a cause of concern. However, most consumers felt that they could make decisions to avoid unsafe beef. In general, consumers felt beef was safest when purchased raw and cooked at home, so that one could control the cooking process and avoid contamination during or after. Signs noted for recognizing unsafe beef (in descending order of frequency of mention) included the color (looking green, brownish, or dark, compared to red); bad smell; presence of flies at the purchase point/on the meat; presence of dust; unclean vendor; type of tables used or their cleanliness; use of an official abattoir; and taste. Two consumers noted that keeping meat packaged in plastic for a long time could lead it to spoil, and a couple respondents noted that the use of trusted vendors would help them to avoid unsafe beef.

*Yes, and when I noticed a food vendor is very neat, and always drives away dust from his meat, then it shows that the meat will be good…. I will take a good look at the meat seller to know if he is neat, and I will also look at the environment, then the way the meat is being displayed. I take note of all that. But if it happens that I send someone to get it, I will not know anything much, and we may end up just cooking it. Like I said earlier, if I buy from those I trust, I have nothing to worry about.–C1205, a 53-year-old male consumer*

Fish commonly available in Birnin Kebbi include fresh tilapia, mackerel, and catfish, as well as smaller dried fish and fried ready-to-eat fish. For Phase 1 of the study, discussions of fish focused on catfish, which is sold alive and reported to be the most commonly purchased fish. In Phase 2, the discussion broadened to also include fresh tilapia, but this was not very commonly purchased. Fish were associated with a moderate amount of food safety concern in the minds of consumers: considerably less than cowpea or beef, but somewhat more than rice. Of those consumers (approximately 40%) with no concern, several reported fish to be safe because they were “healthy” foods recommended by health personnel for other reasons: “No, I don’t think this fish can harm anyone,” one female consumer explained, “because it is a body-building food and helps prevent heart disease” (C1210, 25 years old). 

However, some respondents noted safety concerns associated with fish: spoilage over time, worms, being ill or dead before sale, and chemicals being used in raising or catching fish. Three-quarters of interviewed consumers felt they could recognize safe fish in the market through various signs (in descending order of frequency of mention): alive or moving fast, the color, the texture (not being sticky or slippery), other aspects of the fish’s appearance (i.e., being swollen), appearance of the vendor and sale environment, taste, and smell. One respondent also mentioned avoiding vendors and buying fish directly from fishermen or fish farmers.

*When I am buying fish, I take note of how they react inside the water. If they move very fast and are lively inside the water, it means such a fish is healthy. But when you notice a very dull fish in the water, it shows that fish is sick, and I don’t go for that fish. And to know a harmful or dead fish even when they have already cut it, there is a foam-like whitish substance that comes out of the mouth. So, when I noticed such, I don’t allow them to sell it to me. There are things I am very careful to note when buying fish.–C1211, a 38-year-old male consumer*

*Some of the fresh fish, when you look at the rubber the fish were inside, the water will be dirty. Some of the vendors keep their fish in rubber with water that is clean. You will see the fish swimming about. Then I select the one that I want. And then the table the vendors use in cutting the fish, some of the vendors will use it to cut their fish and flies will be swarming on the table. While some of the vendors, if they use their table, they make sure that they clean the table. You will not know if they use the table or not. Because it will be clean.–C2212, a 30-year-old female consumer*

It was generally noted that fresh fish were the safest form. Dried and fried fish were seen as riskier, due to contamination (e.g., by dirt) after cooking/drying, unsafe cooking practices (e.g., smoking with plastic bags), or because the cooking/drying made it hard to determine the fish’s original type and quality.

Overall, consumers had a moderate level of concern about food safety of GLV, more than fish but less than beef or cowpea. The concerns most commonly mentioned were specifically related to insects or chemicals, with less common mention of washing with unclean water, spoilage, and dirt. Women were particularly likely to note safety concerns related to GLV. All consumers who had concerns about unsafe GLV felt they could recognize which were safe. The main ways of recognizing safe GLV were all based on visual signs: no insect damage (holes) or visible worms/insects, color (green as opposed to yellow or red), otherwise looking ‘fresh’, and not being visibly dirty. A few consumers noted that leaves should be kept fresh with water (without specifying clean water), and two noted that large leaves indicated fertilizer use (and were thus a bad sign). A few respondents noted that using a trusted vendor was a way to avoid unhealthy GLV. While visual signals were widely used, their interpretation differed some across consumers.

*If the vegetable looks good, I will buy it, but if it has patches of holes and insect bites, I won’t buy it. And also the ways I know if they are good or not is from the color: if it is very greenish, then it is good, but if it looks yellowish, then it is bad.–C1205, a 53-year-old male consumer*

*Check if it has holes or not, the size of the leaf, if it is too big it means it has a lot of fertilizer, so it means it doesn’t have the nutrients required… the more attractive it looks, the more problems it has because it means it has a lot of fertilizers.–C2216, a 50-year-old female consumer*

*When the leaf is red in color, the vegetable is bad, but when it is green and fresh it is good. The reason why it turns out to be reddish in color could be as a result of the fertilizers applied…. I can’t really say if the red vegetables can cause harm, but it is better to go for the fresh ones.–C1202, a 24-year-old female consumer*

In Phase 1, when cleanliness was not explicitly mentioned by the interviewer, only one consumer noted that it was important to have a clean vending environment for GLV, but when asked directly in Phase 2, many consumers noted paying attention to the cleanliness of the environment and/or vendor, or their hygiene-related practices. Markers that were considered included: the vegetables themselves being clean or washed, well-arranged, covered, or not on the ground; the vendor being clean (including his/her clothes); and the table or setting being clean.

*[When buying GLV, one should] check the neatness of the environment and the person you’re buying from… The table that they put the vegetables [needs] to be clean, and they should use a sack to cover the vegetables; at least they should cover it from all the dust that carries all these diseases, and it will maintain the green leaves from morning till evening.–C2211, a 40-year-old female consumer*

*[One] should look at how well arranged the table is: they should not just join lettuce and spinach and moringa in one place, the moringa should be in its own place, the lettuce, and the remaining vegetables.–C2202, a 31-year-old male consumer*

Consumers also noted that cooked vegetables were generally safer than those eaten raw, and one noted specifically avoiding any that had been pre-cut or prepared in the market, as they were likely to cause diarrhea.

#### 3.2.3. Coping with Unsafe Foods

For GLV, fish, and beef, possible coping mechanisms for potentially unsafe food were examined—i.e., if the respondent bought the food at the market and worried it might be unsafe, was there anything they could do to it at home that might make it safer? About a quarter of respondents felt there was nothing that they could do—they would just throw it out after purchase (or, in one case, eat it anyway and take the chance). Among the majority who felt they did have coping strategies for unsafe foods, those named included (applicable for all three foods unless otherwise noted) washing with water, washing with salt, boiling (sometimes including discarding the water and re-boiling), and (for beef) cooking with certain seasonings. Two noted washing GLV and/or fish with vinegar and one noted washing GLV with commercial “vegetable wash” products.

*For me, I normally use salt to rinse the beef before boiling it. I rinse it like 2 to 3 times. After rinsing it, I add Maggi seasoning, curry, thyme, etc., to reduce the smell of the meat, if it has any, and that’s it, it becomes safe.–C1216, a 28-year-old female consumer*

*…there’s a way a mother can put in place to reduce the risk of diarrhea, that is, by proper washing of vegetables and fruits. When you buy beef from the market, the first thing when you come back is to wash using vinegar.… it kills unseen germs, that’s what vinegar is used for; in a place where there is no vinegar, you use salt.…–C2210, a 35-year-old female consumer*

In general, women appeared more likely to name different strategies for coping with unsafe foods than men. While coping mechanisms for unsafe grains/legumes were not examined in detail, respondents noted various home-based practices, such as carefully washing rice to remove stones, freezing cowpea to kill weevils, and cooking beans/rice with potash. 

When asked about ways to prevent diarrhea or foodborne illness (not necessarily specifically related to food), participants noted washing food (5 of 31 consumers), avoiding particular foods or eating less of them (3), buying from a clean shop/vendor (3), handwashing or personal hygiene (2), cooking food well (2), and covering food (1).

#### 3.2.4. Learning about Food Safety

Most consumers mentioned having previously heard information or advice about food safety. The main sources mentioned were friends or neighbors (10 out of 14 people), parents (4) and other relatives (6), radio (6), television (5), and doctors or health workers (5). Less commonly cited sources included district government officials, text messages, and women’s organizations. Men generally named more information sources than women and were more likely to name friends/neighbors and radio, whereas women were more likely to mention parents. When asked to whom they would turn for advice on food safety issues, relatives were most commonly named, followed by doctors and health workers, friends/neighbors, and market or environmental officials. Nearly all households had TVs and mobile phones, whereas only about half had radios; radio was thus a comparatively commonly cited information source, whereas mobile phone messaging was rarely cited, despite widespread phone ownership. Messages heard by more than one person included the importance of washing food before preparing, having a clean setting for cooking/food preparation, avoiding chemicals, washing hands or pots, and avoiding unclean food or water. Consumers often expressed limited confidence in the information they had heard.

*…on radio and television, they usually talk about food hygiene, environmental cleanliness, and the need for pregnant women to always eat body-building food… I also get informed from friends, but one cannot verify the information, if it is true or false. We only accept it as it is…. There was a time I was told that there are beans with chemicals that can cause harm to the body. When I heard about it, I stopped buying beans so as not to fall a victim.–C1205, a 53-year-old male consumer*

*I heard that eating unhealthy fish can cause bodily harm on a radio show. They informed us that eating an unhealthy fish will make one fall sick. But I don’t even know how to identify unhealthy fish, so all that we are discussion here now is just my personal thoughts…I may be wrong please, so you don’t say I’m misleading you.–C1214, a 30-year-old man*

### 3.3. How Do Vendors Identify and Mitigate Food Safety Risks?

#### 3.3.1. Recognizing Unsafe Food

Cowpea. The cowpea vendor interviewed generally aligned with the interviewed consumers in terms of how he would identify safe cowpea, but with more detailed techniques: absence of weevils was detected by lack of holes and visible weevils/eggs; it was also noted that cowpea infested with weevils would be warm to the touch, indicating it was unsafe. Chemicals were detected by smell—as a cowpea vendor noted, “nobody will come to buy beans without smelling it” (V1108, 65-year-old cowpea vendor). Avoidance was also a strategy used:

*Yes, [consumers complain about] beans [cowpea], once they discover it has pests, they complain. Me, I hardly [ever] buy beans that have pests, I prefer not having [them] than to have the ones that has pests. So, they complain about that.–V1110, a 38-year-old grain and legume vendor*

Rice. As for consumers, the main issue vendors noted in connection with rice was the presence of stones. Consumers were said to ask about and look to see this when deciding whether to buy. To determine quality/safety, rice was inspected to be clean, free of chaff, and free of stones. 

Maize. For maize, vendors had fairly little concern about quality or safety issues (as in the consumer interviews); three vendors specifically noted that maize was safe because no chemicals were used on it. The main issues that were named were dirt and remainders of the cob left in. Detection of such issues was also visual and entailed ensuring a strong unbroken bag, no visible dirt, and it being dry. No respondents mentioned aflatoxins. 

Soybean. Soybeans were also not a main source of vendor concern related to quality and safety; indeed, it was noted that they were less prone to pests than cowpea, which was an advantage to selling them even though they had a fairly small market. In addition to stones, vendors noted unripe or spoiled beans, and dirt or sand.

*[Consumers’ second-most-common complaint is] sorghum that has stones, and soybeans…. When I get complaints like that … what I do is after coming back from where I buy, outside Kebbi, we get laborers to select the stones out of the grains…. I will tell [customers] I am sorry; it is something that will not happen again, and that you are trying your best to bring something good for sale in the market.–V1110, a 38-year-old grain and legume vendor*

For beef, problems identified were sick animals, spoilage (due to lack of refrigeration, a common constraint noted by vendors), use of animal medications, and presence of dirt or flies on the meat. Quality/safety of meat was usually determined by visual inspection: to see that it had a good color (red, not too dark) and was not too bloody. A firm texture and lack of odor were also noted, as was asking the supplier if the animal was healthy. Unlike for the other focus foods, two beef vendors noted the importance of a clean sale environment and vendor in communicating safety/quality and encouraging customers to purchase.

*If you discover that the meat looks soft, honestly the meat is not safe to eat. But if the meat looks high quality…just take a look at that meat, is there any way you can say that you didn’t enjoy the meat? Is there a way they will give you this meat and you will say you didn’t enjoy eating it? You will enjoy it because you know the quality… But if the animal is sick…it will look like this…not attractive.–V1105, a 35-year-old beef vendor*

*Meat with dirt is not healthy … because the business requires neatness. From where we buy up to where we sell, needs to be clean. It’s not healthy if the color changes: maybe it’s left-over meat and the person did not take good care of the meat, the color will be kind of dark.–V1112, a 25-year-old beef vendor*

Vendors of fresh products (as opposed to grains/legumes) generally construed food safety in general terms as related to “freshness”. Risk appeared to be assessed along a continuum of freshness, with the problem of safety arising only at the outer extreme; at intermediate stages, declining quality or freshness was more of a business concern to vendors than a food safety issue per se. 

**Figure 1:**
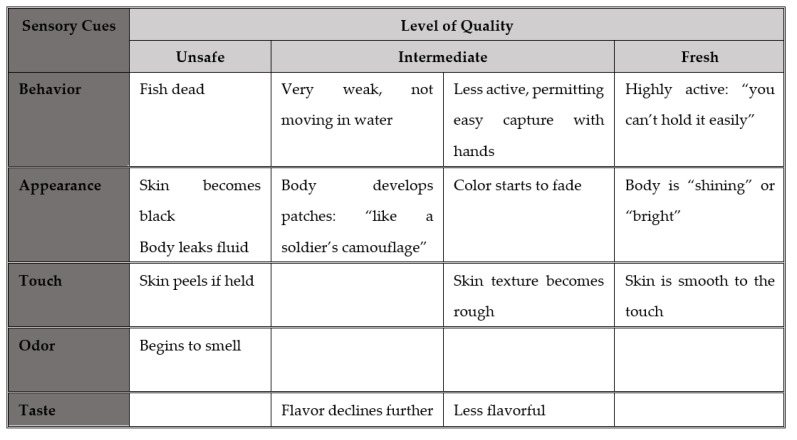
Vendors’ typology of fish quality.

**Figure 2:**
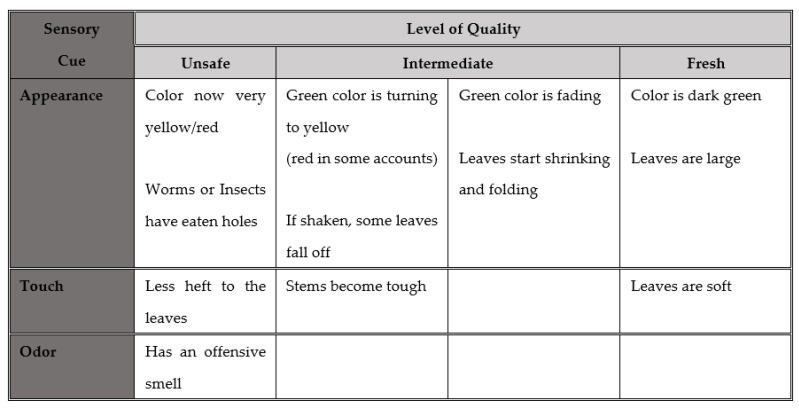
Vendors’ typology of GLV quality.

To explore this, we engaged Phase 2 vendors in an exercise intended to identify commonly recognized points along a food quality continuum, focusing in depth on two foods: fish and GLV. Vendors were presented with a board divided into four sections, representing levels of food quality (best at one end, unsafe at the other, and two intermediate levels in between). Vendors were asked to describe signs that would be evident in fish or GLV at each of the four levels, and these data were synthesized to create a continuum of commonly recognized signs of quality for each focus food (Figure 1 and Figure 2). Several sensory cues were employed in vendor assessments of quality: changes in appearance and texture, and in the final stage, odor. Fish vendors also drew on the behavior of live fish for quality cues.

#### 3.3.2. Avoiding and Mitigating Food Safety and Quality Issues

While vendors could sell some foods that were not fully fresh, once food became unsafe, vendors’ only recourse was to dispose of it. Fish and especially GLV vendors thus considered themselves to be on a “ticking clock” from the moment they received their product from suppliers: without access to refrigeration, the transition from peak freshness to unsalable product can occur within a day or even hours. GLV in particular begins its decline within minutes of harvest. The risk of being left holding product that is less-than-fresh (even inedible) was a preoccupation for vendors.

*There are challenges we face from this business. An example is, when every fish you have is dead, you will end up selling them for even less than you bought them. People love buying fish that are killed in their presence. But when you have fish that are already dead, you will have to convince someone to buy the fish and give the person confidence that the fish is still in good condition.–V2115, a 40-year-old fish vendor*

*Seriously, I really used to make a profit in this business, and I’m proud of the business I am into. But there are times you enjoy the business—* *that is when you sell and make profit—* *and times you will not want to do this business, especially during heat. It’s hard to maintain the freshness of the vegetables because of the hotness of the weather.–V2121, a 25-year-old GLV vendor*

To slow the decline in quality, vendors drew on a limited repertoire of actions. Fish vendors ensured that the fish’s water was changed regularly and that they were protected from the sun during transport; few described other actions (e.g., feeding or care, temperature conditions, oxygenation). GLV vendors described cutting the roots from fresh greens after they received them from suppliers, washing any sand from the leaves, covering them with a sack to protect them from drying winds and sun, and periodically sprinkling with water to keep their appearance fresh. 

The choice of suppliers could also play a role in mitigating potential safety or quality issues. Vendors noted both using preferred, trusted suppliers and “shopping around” in a wholesale market to find the best quality-price combination. The main driver of choice named when it came to choosing a supplier was high-quality products, followed by affordable prices and the ability to buy on credit. Two vendors mentioned seeking a trustworthy or honest supplier, which was also described in terms of having a quality product and being truthful about it and being honest in pricing.

*What is important to consider during business is that I look for a healthy animal, that I won’t be doubting when buying it. And, again, if I come to where to sell it, I will still check if the animal is healthy before slaughtering it, because if it’s not healthy, then there will be plenty of trouble, and I don’t want that.–V1112, a 25-year-old beef vendor*

*If I know my fish is not healthy or good, I worry that customers will not use it and [will] start complaining. If I see any sign of fish that has a problem, I do not buy… I prefer not to buy to harming shoppers.–V1111, a 55-year-old fish vendor*

Vendors generally felt comfortable discussing quality issues with suppliers and trusted that suppliers would be honest in their reports on food quality, or that it would be clear from a visual inspection. Fish and beef vendors in particular noted that they would search for healthy animals and inquire about that from the supplier. The main reported reaction to a poor-quality product was simply to not buy it and leave the market empty handed or, if the problem was discovered after purchase, to not patronize that supplier again.

*If I discover that the meat is not good for consumption, I will not buy it… I choose good meat so that I will be able to sell good meat to my customers, so that they will visit me again. I would not like to sell bad meat to my customers. It will scare them away, and they will not come back to me again tomorrow… There are people who [buy potentially harmful meat], but I don’t.–V1105, a 35-year-old beef vendor*

### 3.4. Who Is Seen as Responsible for Food Safety?

Asked to consider who ought to be taking charge of food safety, most vendors (8 of 13) proposed some arm of government, including health personnel and city officials. Other suggestions included market leaders and suppliers. Only two vendors initially noted that some responsibility rests with vendors themselves, but following prompts from the interviewer, five vendors acknowledged that vendors could also play a role. Similarly, most consumers (and all but one male consumer) referred to the government, and five more specifically mentioned health officials. Beyond government, consumers interviewed mentioned market management/officials (3 of 16 respondents), vendors (3), suppliers/farmers (3), consumers themselves (2), security officers (1), and traditional leaders (1).

Only some consumers thus held vendors responsible for ensuring food was safe. While most consumers opined that the food they were getting from vendors was safe, most also felt that, were a vendor to be in possession of unsafe food, he/she would be unlikely to tell potential customers. Consumers recognized two sources of risks associated with vendors: some vendors were dishonest and would knowingly prioritize profits over quality/safety; others were simply unaware.

As noted on the subject of avoiding unsafe food, some consumers seemed to feel a lack of agency when selecting a vendor who could guarantee safe food:

*Well, they [vendors] can sell anything they like, because we are not the sellers, we only buy from them. You know a vendor will never bring beef to the market and say the beef he has for sale is unsafe. They can only say it is good… They can’t tell, since they’re in a business, everyone wants to sell their products, so they will never tell the consumer if anything is wrong with it.–C1201, a 30-year-old female consumer*

*Well, I don’t think they [vendors] know [whether the fish they are selling is safe]. They don’t because most that sells fish, they are…permit me to use the word, illiterates. And their eyes are closed. Their own is just for them to look for money. So, they don’t care if it is safe or unsafe for the consumers.–C1209, a 40-year-old male consumer*

*Sometimes you get good ones, sometimes you get not-too-good ones. For example, old [beef] stock that has been kept in the fridge overnight and brought out in the morning to be sold as fresh beef. Very bad. The same with killing of sick animals. The sickly animals are slaughtered because the meat sellers want to make money while no one is considering the people that will buy it to eat.–C2204, a 40-year-old female consumer*

Asked what vendors could do to reduce food safety risks, consumer respondents generally noted specific actions like washing or covering food, using fewer chemicals, not storing food on the ground, sourcing high-quality products, or keeping themselves and/or their selling environment clean. Additionally, involved authorities were named (particularly to undertake inspections), engaging the vendors’ union, providing training, having certified vendors, having a complaints/reporting mechanism for vendors who sell unsafe food, and/or promoting cleanliness. Three respondents also noted the need to provide refrigeration.

*If they [vendors] can reduce the way the flies perch on the vegetables it will be very good… Because you don’t know where the flies had perched, and they will stay on the vegetables and people will come and buy…. They should make sure the place is not smelly and it is clean.–C2201, a 25-year-old male consumer*

*Vendors should by all means make sure they slaughter the animals [cows] themselves. Just like they do in other developed countries, the government should make sure there are teams of inspectors who inspect the abattoir daily to make sure what they are selling is safe for consumption.–C1209, a 40-year-old male consumer*

## 4. Discussion

This paper used detailed data from interviews with consumers and vendors in three traditional markets in Birnin Kebbi, Nigeria, as well as observations from those markets, to examine how consumers and vendors identify and avoid or manage food safety risks and whom they hold responsible and trust when it comes to ensuring food safety. The research provided novel insights into consumer and vendor decision-making in a context where food sold in local markets cannot be assumed to be safe. Considering the first question, consumers deployed two main strategies to decrease the risk of purchasing unsafe food: choosing the right vendor and choosing the right food. As noted in a study in Vietnam, the choice of market was largely not influenced by food safety concerns, and was instead routinized [19]. At the level of the vendor, however, safety factors did play a role in that many consumers mentioned seeking “clean” or “neat” vendors or stalls. Cleanliness was primarily related to the appearance of the vendor, stall, and surroundings; it included aspects likely to be correlated to food safety (e.g., not placing goods on the floor) but also those not (e.g., neatly piled goods). A similar prioritization of aesthetic appearances of cleanliness—i.e., the social performance of safety—was found among street vendors and consumers in Ghana [31]. A focus on cleanliness as a marker of health has also been noted in studies of consumers in Ethiopia, South Africa, and Brazil [32,33,34].

Comparing to existing guidance on best practices for food safety in traditional markets [35], some elements are mentioned (e.g., vendors wearing clean clothes, waste managed, separating products, vendor’s observable hygiene); others are not or rarely so (e.g., cold storage, use of clean water, short fingernails, avoiding eating/smoking, handwashing). This offers an interesting contrast to findings from cross-sectional “knowledge, attitude, and practice” surveys from Nigeria, which tend to ask directed questions on these expert-identified best-practices, and in response often find relatively high levels of knowledge and self-reported best practices along these dimensions [21]. Of note, certain hazards like aflatoxin, known to be present in maize and other foods in Nigeria [36,37], were never mentioned, and neither vendors nor consumers had any strategies to detect or avoid them. 

Reliance on trusted, known vendors was also noted, particularly for goods for which quality was difficult to determine via inspection, like stone-free rice. This aligns to findings from Vietnam [19,38], Ghana [31], and Rwanda [20] and suggests a potential economic benefit to vendors from ensuring safe food. However, consumers made almost no mention of traceability, local sourcing, or asking about food’s origin—in contrast to results found in Vietnam [19,38,39,40] and high-income countries [19,41,42,43,44]. Use of informal networks or “buyers’ clubs” were also not mentioned, though such practices have been noted in Russia and Vietnam [39,45]. Use of formal certifications were also not mentioned, which is unsurprising as such certifications do not exist for traditional market vendors in Kebbi; such approaches have been used with success in traditional market settings elsewhere [46], though there is doubt regarding the long-term effectiveness of labelling-based approaches [47,48].

For food products themselves, these were largely evaluated based on visual cues: insects, holes, and colors, with some reliance on smell, also. Similarly, vendors assessed safety of food from suppliers based on a visual assessment or reliance on trusted relationships. While research in Kenya has found vendors to have different food safety perceptions than consumers [49], here the cues used were largely similar across the two groups. Among both, safety was generally associated with visually apparent freshness, as noted in research in South Africa and Italy [33,43].

Turning to the second research question, both consumers and vendors largely placed responsibility for ensuring food safety on government. Nigeria has adopted multiple food safety-related policies covering multiple stages of both domestic and export value chains [13]. The main policy governing food safety is the National Policy on Food Safety (2014) [50]; it outlines a food safety mandate spread among 13 ministries, departments, and agencies and creates a national Food Safety Management Committee. However, the bill supporting the policy’s implementation has not yet been enacted into law, and many elements of the policy have been only partially implemented and enforced [13,18]. Inspections and enforcement rarely reach to secondary cities such as Birnin Kebbi [31]. While consumers did not often name vendors as among the main parties responsible for food safety, when asked specifically they did name actions that vendors could take to ensure food safety. However, none mentioned communicating with vendors about these actions. Indeed, two opined that doing so would not be appropriate or would not be “their place”. This reticence may be due to bartering and a desire to achieve a lower price.

The lack of perceived mutual accountability also somewhat contrasts with strong agency in other areas of perceived risk control. With some exceptions, consumers and vendors generally felt that they could limit many food safety risks through identifying the “good” products in the market or from suppliers and, in the case of consumers, mitigating risk through in-home practices. This was in some contrast to consumers’ views on vendors: a significant share of consumers felt they had little ability to ensure the veracity of a vendor’s claims and might well be being sold unsafe or poor-quality products—similar to findings for street food sellers in Ghana [31]. Vendors also agreed that at least some vendors sell unsafe or poor-quality food, even if they themselves opined that they never would. Vendors and consumers thus feel agency in some areas of avoiding food safety risk, but not others, and in many cases place the responsibility for controlling it in other hands. A similar lack of perceived responsibility for food safety risks, or agency to control them, has been found among consumers in Brazil, South Africa, and, for chemical hazards, several high-income countries [33,41,51] and among vendors in Vietnam [19]. Leveraging consumer demand to drive safer food has been argued to be an important way to improve safety in transitioning LMICs and was a major historical driver of safer food in middle- and high-income countries, such as the U.S. [52,53]. Doing so in Birnin Kebbi, however, will require convincing consumers that they play an essential role in keeping supply chain actors and other decision makers accountable for food safety in the market itself, and that there are specific, actionable steps they can take to achieve it (such as communicating with vendors). 

The study had certain limitations. The research focused primarily on seven foods; while these span food groups and represent many foods commonly eaten in Nigeria, some (e.g., fruit) are omitted. The sample was small and not fully representative of the local population. In particular, no female vendors were identified as eligible for interviewing, and the consumer sample was relatively affluent and well-educated, compared to the average for urban Nigeria. The study results would thus benefit from being examined additionally through larger-sample, survey-type research.

## 5. Conclusions

We conclude by drawing on the study results to offer suggestions for improving food safety in Nigerian markets. Given that, for consumers, food safety was most relevant when it came to selecting a vendor (as opposed to a market), leveraging consumer demand to improve vendors’ food safety behavior is promising. This could be done, for example, through a “clean vendor” certification approach (as used, e.g., in India [46]). To be impactful, certification would need to be tied to actual correlates of food safety, not “performative” aspects of neatness. There is some evidence that Nigerian consumers would be willing to pay more for safe-certified food, though this may not extend to all product types, particularly for low-income consumers [54]. Hence, such an approach should not be the only one used, or it could lead to inequitable outcomes for consumers. 

There may also be a role for educating consumers on signs that vendors are following “best practices” for market food safety, as several of these were never mentioned. Vendors and consumers will likely be more responsive to interventions that focus on issues of freshness and quality, rather than food safety per se: both consumers and vendors tend to speak of quality/freshness and safety somewhat interchangeably, or as occurring along a continuum. For vendors of perishable foods, their key challenge is how to obtain a profit for goods that are losing value with each hour. They are likely to see value in any intervention that can change the equation, whether on the supply side through improving transport or storage conditions, or on the demand side, for instance by using new approaches to expand customer reach. For consumers, freshness is already valued, and building on that with a more in-depth understanding of spoilage, contamination, and their consequences could help ensure a closer correlation between the types of cues they use to make decisions on food safety and actual markers of food safety risk.

Both consumers and vendors placed primary responsibility for ensuring food safety on the government. As noted above, however, there are various weaknesses in existing Nigerian food safety policy and enforcement. Based on prior analyses [13,18], key opportunities for strengthening the national food safety system include building local capacity for collection, analysis, and timely communication of food safety and foodborne disease occurrence data, developing systems to respond to disease outbreaks, and increasing the capacity of the inspection system. The present study emphasized that such changes should be designed and implemented in a way that meets the needs of not only formal, well-developed value chains, but also the type of traditional, locally oriented markets studied here.

In summary, this study has made it clear that vendors and consumers in Birnin Kebbi deploy a range of strategies to detect and mitigate potential food safety risks. While some of these do not align with scientific evidence on causes of food safety risk, many do, and they constitute a solid foundation of techniques to build upon with future interventions. At the same time, consumers and vendors largely place responsibility for food safety on government, not themselves, and have conflicting views of their personal agency in ensuring safe food. Interventions will thus need to focus not only on practical steps that can be taken to reduce food safety risk, but also on convincing vendors and consumers of the need, and their ability, to act to increase mutual accountability across food system actors. 

## Figures and Tables

**Table 1:** Consumer demographic characteristics (*n* = 47).

**Respondent Characteristics**	
Gender	Male (49%), female (51%)
Average age (range)	33.7 (22–64)
Ethnicity	Hausa (47%), Zuru (30%), Fulani (15%), Igbo (6%), Other (9%)
Religion	Muslim (62%), Christian (38%)
Highest education completed	Primary (94%), Tertiary (53%)
Marital status	Married (monogamous)—66%; married (polygamous)—6%; single—26%, widowed—2%
Principal household income earner	45%
Occupation	Professional/Managerial—30%; Small business owner/entrepreneur—15%; Not employed outside home—23%; Sales/services employee—11%; Petty trader, hawker—6%; unskilled labor—2%, technical labor—9%, agriculture—4%
**Household Characteristics**	
Avg. household size (range)	6.2 (1–19)
Avg. number of children (range)	2.6 (0–11)
Home has electricity	91%
Pct. poor (1.90 PPP) *	2%
Pct. poor (3.10 PPP) *	12%
Household owns car	32%
Household owns mobile phone	98%
Household has improved toilet	91%
Farms or owns farmland	55%

* Note: reproduced from [27].

**Table 2:** Vendor demographic characteristics (*n* = 37).

Vendor Characteristics	
Percent male	100%
Average age (range)	40 (22–65)
Ethnicity	Hausa (95%), Fulani (5%)
Religion	Muslim (100%)
Pct. completing primary school	51%
Pct. completing secondary school	22%
Pct. completing tertiary school	3%
Avg. years vending (range)	19.2 (5–43)
Respondent is household’s principal income earner	95%
Respondent has another income source	70%
Other income sources	Farming or livestock (23); selling other food/goods (2); contractor (1)

Note: reproduced from [27].

**Table 3:** Main Market Characteristics.

Market	Estimated Area	Estimated Number of Vendors	Toilet Facilities?	Water Facilities?	Garbage Facilities?
Main city market	3800 m^2^	About 1875; almost 90% male	Yes (commercial and public)	Yes	No
Neighborhood market 1	500 m^2^	About 200; about 80% male	No	No	No
Neighborhood market 2	420 m^2^	About 13; less than 50% female (all are teenagers selling rice)	No, but an incomplete building is used for urinating	No	No
